# Understanding the ramifications of quantitative ordinal scales on accuracy of estimates of disease severity and data analysis in plant pathology

**DOI:** 10.1007/s40858-021-00446-0

**Published:** 2021-07-13

**Authors:** Kuo-Szu Chiang, Clive H. Bock

**Affiliations:** 1 Division of Biometrics, Department of Agronomy, National Chung Hsing University, Taichung, Taiwan 402; 2 USDA-ARS-SEFTNRL, Byron, GA 31008 USA

**Keywords:** Nearest percent estimates, Plant disease assessment, Scale design, Calculation of the interval range

## Abstract

The severity of plant diseases, traditionally defined as the proportion of the plant tissue exhibiting symptoms, is a key quantitative variable to know for many diseases but is prone to error. Plant pathologists face many situations in which the measurement by nearest percent estimates (NPEs) of disease severity is time-consuming or impractical. Moreover, rater NPEs of disease severity are notoriously variable. Therefore, NPEs of disease may be of questionable value if severity cannot be determined accurately and reliably. In such situations, researchers have often used a quantitative ordinal scale of measurement—often alleging the time saved, and the ease with which the scale can be learned. Because quantitative ordinal disease scales lack the resolution of the 0 to 100% scale, they are inherently less accurate. We contend that scale design and structure have ramifications for the resulting analysis of data from the ordinal scale data. To minimize inaccuracy and ensure that there is equivalent statistical power when using quantitative ordinal scale data, design of the scales can be optimized for use in the discipline of plant pathology. In this review, we focus on the nature of quantitative ordinal scales used in plant disease assessment. Subsequently, their application and effects will be discussed. Finally, we will review how to optimize quantitative ordinal scales design to allow sufficient accuracy of estimation while maximizing power for hypothesis testing.

## Introduction

Disease severity (the proportion of a plant unit diseased, Nutter Jr. et al. 1991) data is widely used by plant pathologists for predicting yield loss, monitoring and forecasting epidemics, in disease surveys and for assessing crop germplasm for disease resistance, and for understanding fundamental biological processes including coevolution (Bock et al. 2010b). Therefore, quantitative information on disease severity is paramount. Several scale types are used in visual plant disease assessment (Bock et al. 2010b; Madden et al. 2007), and these include nominal, ordinal, and ratio scales (Stevens 1946). Where disease area (the proportion of the specimen area showing symptoms) is visually estimated by raters, the continuous percentage ratio scale is commonly used (Yadav et al. 2013; Schwanck and Del Ponte 2014). Using the percentage scale, raters estimate the area showing symptoms of disease relative to the whole area of the object (leaf, fruit, *etc.*) to the nearest percentage point, sometimes called the nearest percent estimate (NPE). Almost invariably the estimates will differ from the actual values, due to characteristic absolute error in estimation across the range of disease severity (Kranz 1970; Forbes and Jeger 1987; Bock et al. 2010a). Therefore, using NPE estimates of disease severity may be of questionable value if severity cannot be determined accurately and reliably, or perhaps if there is insufficient time to apply the percentage scale. In such situations, researchers have often used an ordinal scale of measurement.

Ordinal rating scales comprise a characteristic structure of rank-ordered, numeric classes, *i.e.*, 1-to-*n* (*e.g.*, *n* = 5). Considering the structure, ordinal scales may be used to obtain two kinds of measurements. Firstly, they may be quantitative if based on the percentage scale (as is most common), with each class described by consecutively defined ranges of numeric magnitude. An example of a quantitative ordinal scale based on 12 consecutive ranges of the percentage scale is the Horsfall-Barratt scale (Horsfall and Barratt 1945). Secondly, ordinal scales may be qualitative, if based on descriptions of the progress of symptom development. An example of a qualitative ordinal scale is a descriptive, 5-class scale developed to assess the severity of zucchini yellow mosaic virus and watermelon mosaic virus in watermelon by Xu et al. (2004). The severity of disease obtained with a qualitative ordinal rating scale is a rank-ordered numeric variable, but the qualitative ordinal rating scale is based on descriptions of symptoms (Madden et al. 2007; Agresti 2010). It is not statistically appropriate to take means based on these scales (Stevens 1946) as it has little meaning biologically, and violates assumptions underlying parametric tests. Qualitative ordinal scales can be analyzed using non-parametric statistics suitable for various experiment designs and distribution functions (Shah and Madden 2004; Fu et al. 2012). We consider only quantitative ordinal scales here in this review article.

Although the use of quantitative ordinal scales has been described and discussed as a component of broader reviews on plant disease assessment (Chester 1950; Chaube and Singh 1991; Madden et al. 2007; Bock et al. 2010b), there have been some different terms used to discuss quantitative or qualitative ordinal scale data in the plant pathology literature. For example, Fu et al. (2012) used the term “ordinal qualitative data” collected for phenotypical measurements in plant pathology and other biological sciences. Madden et al. (2007) (second paragraph, p20) states that the severity of disease obtained with an ordinal disease rating scale is clearly an ordered categorical variable (referred to as a “qualitative ordinal scale” in this review article), or corresponding to an ordinal measurement level (referred to as a “quantitative ordinal scale” in this review article). Indeed, quantitative ordinal scales have been termed “interval scales” (Nutter Jr. and Esker 2006; Bock et al. 2009a), “ordinal scales” (Hartung and Piepho 2007), “interval” or “category” scales (Bock et al. 2010a), and “category scales” (Chiang et al. 2014). In a plant breeding study (Xie et al. 2012), the term “semiquantitative scale” was used. Due to this confusion of terms, the scales have been recently defined and referred to as “quantitative ordinal scales” (Bock et al. 2016; Chiang et al. 2020). The American Phytopathological Society in its instruction to authors considers them simply an ordinal scale (Anon 2020). We believe that clearly defining quantitative ordinal scales as used in the discipline of plant pathology will help clarify the confusion in terminology. To the best of our knowledge, this is the first review to focus solely on quantitative ordinal scales, to explore their characteristics and mechanics, so as to help guide their development and application in plant pathology, and to avoid poor scale design and misuse in the future.

**Fig. 1:**
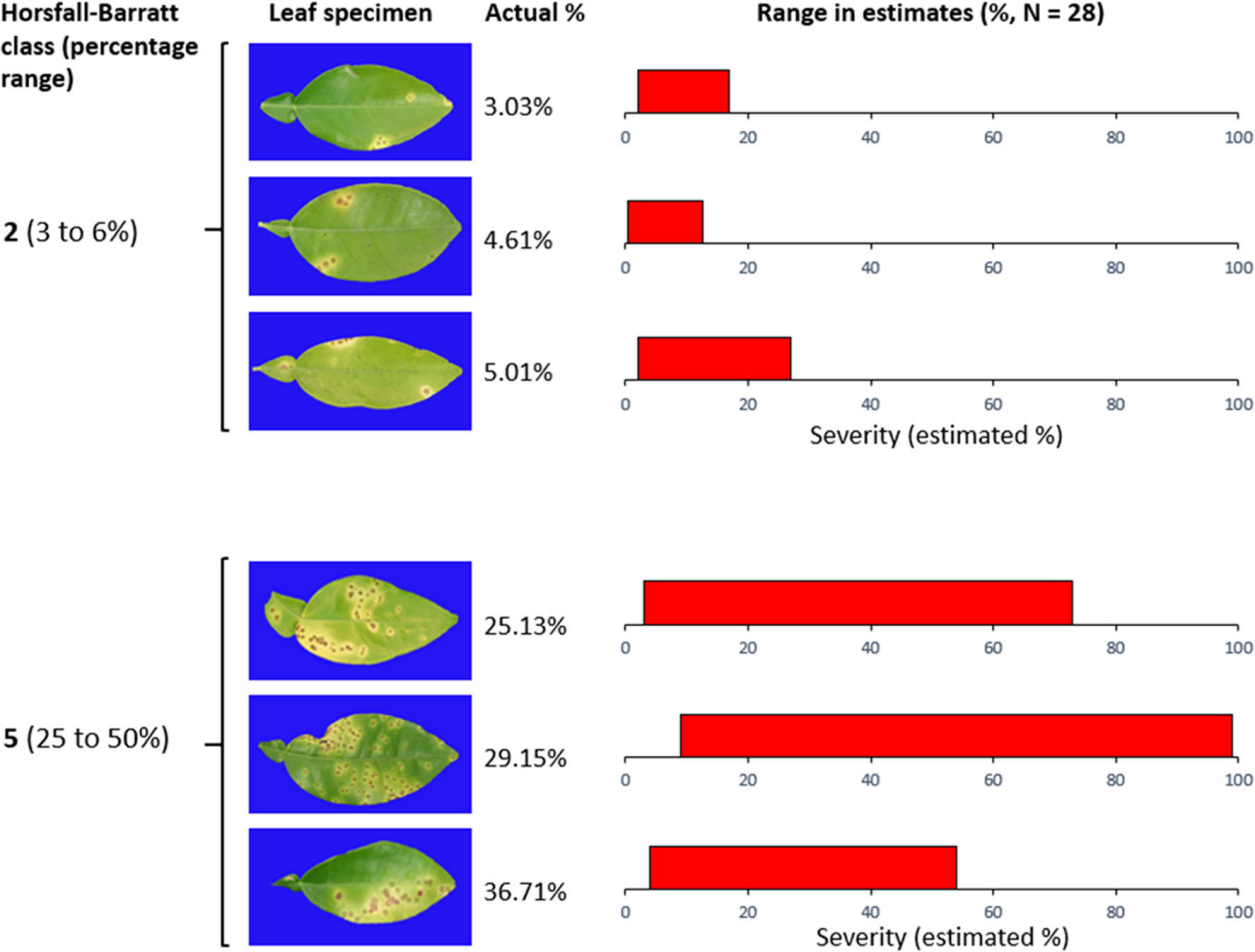
Severity of citrus canker on six grapefruit leaves, three classified in each of class 2 and 5 of the Horsfall-Barratt scale. Estimating the actual value can be challenging depending on the nature of the symptoms, as indicated by the range of estimates by 28 raters of the three leaves with visually similar severity in the two different classes. Note that at severities in class 5, the range of estimates spans a much wider percentage. In this case, using image analysis (Assess®; Lamari 2002), the severity was measured and is indicated as the actual value, which can be considered accurate when taking into account the chlorotic and necrotic areas. Using the ‘Chiang’ scale the leaves would be classified in class 5, 6, 8, and 9, depending on the particular specimen (Table 1)

As noted, a reason for choosing a quantitative ordinal scale may be for convenience and speed of rating (Madden et al. 2007). Another reason is that a rater may not be capable of easily distinguishing differences in severity within an ordinal class (see Fig. 1). However, to be useful, disease severity estimates using a quantitative ordinal scale must be accurate and reliable. Here accuracy is defined, as it is in measurement science, as the closeness of the estimate to the true value, and reliability is the extent to which the same estimate obtained under different conditions yields similar results (Madden et al. 2007). For sampling and data analysis, the accuracy of the mean value of all specimen estimates relates to the deviations between the estimated mean (sample mean) of the variable and the actual mean value of that variable in the population (Madden et al. 2007). The individual specimen estimates are used to calculate the mean severity of the sample in the population, which in turn is often used to compare treatments. Thus, although they are different aspects of disease assessment, accuracy of the individual specimen estimates and the mean estimates are inextricably linked. The accuracy of the mean value is dependent not only on the accuracy of estimated values (as in measurement science) but also on other criteria including the interrelated factors of sample size, sampling strategy, and spatial distribution of the disease (Bock et al. 2016; Madden et al. 2007). If inaccurate or unreliable disease assessments are obtained, this might lead to faulty conclusions being drawn from the data, which in turn might lead to incorrect actions being taken in disease management (Bock et al. 2010b).

Certain characteristics of a quantitative ordinal scale design may affect the accuracy of the specimen estimates and, consequently, affect the accuracy of the resulting mean disease severity for the sample. The reason is that additional variability may be added into the samples due to the structure of some ordinal scales (Chiang et al. 2014). It is important to know the extent to which this information is distorted, and the impact it has on the accuracy of the mean value and variance of the sample mean. If the distortion is severe, it will result in inaccurate mean estimates and could affect the outcome of an analysis. Inaccurate estimates of mean disease severity could have practical consequences when these estimates are used as predictors of crop loss, differentiating treatments, or for other decision-making purposes (Madden et al. 2007). The effect of scale structure must be understood before steps can be taken by practitioners in plant pathology to optimize the designs of quantitative ordinal scales in situations where the scales are preferred.

The objective of this review is to provide the reader with a more extensive consideration of quantitative ordinal scales. First, we provide a brief review of the concept of these scales (at least those used in plant pathology). Second, we describe the ramifications of their use based on both empirical and simulation-based studies. Third, we discuss ways to design quantitative ordinal scales to best minimize bias in estimation and maximize the statistical power in hypothesis testing. Fourth, we describe briefly their use as surrogates for “actual values” for ground-truthing fully automated sensor-based measurements of disease severity. Finally, some suggestions on how best to use the scales in relation to the planned analysis of the data are provided.

## An overview of quantitative ordinal scales

### Characteristics of quantitative ordinal scales

With plant disease severity, this special form of the ordinal scale is generally based on the percent area with symptoms. As noted, probably the most well-known example is the Horsfall-Barratt (HB) scale, which divides the percent scale into 12 consecutive and roughly logarithmic-based intervals of severity between 0 and 100% (Horsfall and Barratt 1945). The consecutive intervals increase in size from 0 to 50% and decline in size from 50 to 100%. The structure of the HB scale is presented (Table 1). The HB scale was developed in the early 1940s as the science of plant pathology was becoming more quantitative, and is based on two assumptions. The first is that there is a logarithmic relationship between the intensity of a stimulus (*i.e.*, reflected light from a diseased specimen) and sensation (*i.e.*, estimated area diseased). The second assumption was that when observing an object consisting of two colors or forms, a rater focuses on the one that is smaller in size (Madden et al. 2007). The problem is that there is little experimental evidence that the assumptions behind the HB scale hold for assessing disease severity; all evidence to date shows a linear relationship between visually estimated and actual disease severity, although a nonlinear relation is expected on the basis of the HB scale (Sherwood et al. 1983; Forbes and Korva 1994; Nita et al. 2003; Nutter Jr. and Esker 2006; Bock et al. 2008a, 2010b).

**Table 1:** The HB scale and some other quantitative ordinal scales used as tools for assessing plant disease severity

HB scale (1945)			Chiang et al. (2014)			Xie et al. (2012)^a^		Todd and Kommedahl (1994) ^a^		Poland and Nelson (2011)^b^	
Ordinal equivalent	Severity (% range)	Midpoint	Ordinal equivalent	Severity (% range)	Midpoint	Ordinal equivalent	Severity (% range)	Ordinal equivalent	Severity (% range)	Ordinal equivalent	Severity (% range)
0	0	0	0	–	–	0	0	0	0	0	0
1	0^+^−3	1.5	1	0^+^ − 0.1	0.05	1	1–10	1	1–25	1	0^+^−1
2	3^+^−6	4.5	2	0.1^+^ − 0.5	0.30	2	11–30	2	26–50	2	1^+^−2
3	6^+^−12	9.0	3	0.5^+^ − 1.0	0.75	3	31–50	3	51–75	3	2^+^−5
4	12^+^−25	18.5	4	1.0^+^ − 2.0	1.50	4	51–80	4	76–100	4	5^+^−8
5	25^+^−50	37.5	5	2.0^+^ − 5.0	3.50	5	80–100			5	8^+^−12
6	50^+^−75	62.5	6	5.0^+^ − 10.0	7.50					6	12^+^−20
7	75^+^−88	81.5	7	10.0^+^ − 20.0	15.0					7	20^+^−33
8	88^+^−94	91.0	8	20.0^+^ − 30.0	25.0					8	33^+^−66
9	94^+^−97	95.5	9	30.0^+^ − 40.0	35.0					9	66^+^−100
10	97^+^−100	98.5	10	40.0^+^ − 50.0	45.0						
11	100	100	11	50.0^+^ − 60.0	55.0						
			12	60.0^+^ − 70.0	65.0						
			13	70.0^+^ − 80.0	75.0						
			14	80.0^+^ − 90.0	85.0						
			15	90.0^+^ − 100.0	95.0						

^a^The studies of Xie et al. (2012) and Todd and Kommedahl (1994) did not use the midpoints for computation

^b^The studies of Poland and Nelson (2011) described the disease severity classes with additional descriptors

Several quantitative ordinal scales have been developed that subdivide the percent scale into different numbers of classes and varying interval sizes (Bardsley and Ngugi 2013; Forbes and Korva 1994; Hunter and Roberts 1978; Hunter 1983; Hartung and Piepho 2007; Chiang et al. 2014). Analysis of data obtained from rating using quantitative ordinal scales may be done through mid-point conversion and subsequent parametric analysis, or direct analysis of the class values by using a proportional odds model (Chiang et al. 2020).

Based on the nature of the data, scales are classified as being nominal, ordinal, interval, or ratio (Stevens 1946), where there is increasing information in the data. Many texts (*e.g.*, Freund and Perles 2007; Madden et al. 2007) have also used this definition. In Measurement Science, one of the definitions of an interval scale is that it lacks a true zero (Stevens 1946), while ordinal scales measuring disease severity in plant pathology have a true zero (the state of no disease = healthy). There is more information describing each class using quantitative ordinal scales as compared with most ordinal scales because the consecutive ranked classes are based on consecutive ranges of the percentage (0 to 100) ratio scale, or ranges of a proportion (0 to 1) (ordinal scales in Stevens system of measurement scales are generally equivalent to qualitative ordinal scales in this article). For example, “4” of HB scale represents a range 12^+^ to 25% severity. Thus, a quantitative ordinal scale contains more information than qualitative ordinal scales. Quantitative ordinal scales rarely have equal differences between scale classes (and thus as a further generalization they are not interval scales). So, we contend that the umbrella term “quantitative ordinal scale” for ordinal scales with numeric intervals or “ranges” is appropriate.

### Errors in estimation when using quantitative ordinal scales

Quantitative ordinal scale design could introduce additional errors besides rater bias (Forbes and Korva 1994; Nita et al. 2003; Bock et al. 2009a). Here, error refers to bias around an actual value. At what ranges of actual disease severity are the greatest errors of individual estimates? In fact, the characteristics of error estimation can depend on whether the actual disease is compared with one individual’s estimates, raters as a group, or the mean values. It may also depend on the time taken to perform an assessment, and the use of aids including standard area diagrams, training, instruction, and experience. In some studies where multiple individual rater estimates were used, it was demonstrated that there is an increase in error of estimates with increasing actual disease severity (Koch and Hau 1980; Forbes and Jeger 1987; Hau et al. 1989; Bock et al. 2008b, 2009b; Franceschi et al. 2020). Other data based on well-trained or instructed individuals using an assessment aid or on mean values might be less likely to show increased estimation error or heterogeneity of variance due to actual disease severity (Nita et al. 2003; Nutter Jr. and Esker 2006). Moreover, some studies have found a hyperbolic (Bock et al. 2010a; Chiang et al. 2016a) or a quadratic linear relationship (Franceschi et al. 2020) between the standard deviation of the mean estimate and the actual value, indicating a pattern in magnitude of absolute errors. Thus, the nature of the relationship between the variance of the mean estimate and the actual disease is not been completely understood (Bock et al. 2010b).

### Effects of scale interval and structure

**Fig. 2:**
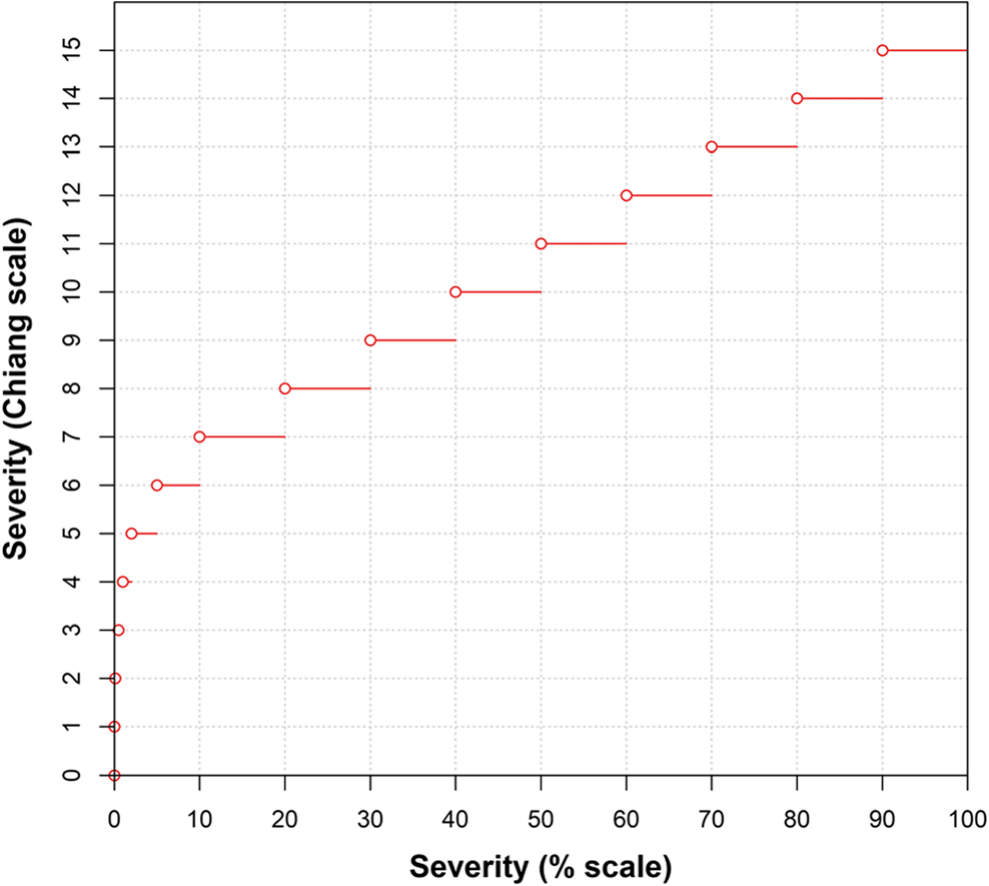
The relationship between the ordinal class using the “Chiang” scale (2014) and the actual percent severity on a sample

The number of classes in an ordinal scale has been discussed by Kranz (1970, 1988) and Hau et al. (1989) in relation to scales with unequal-sized categories. Kranz (1970, 1988) suggested that approximately seven classes (in addition to zero) were optimal for division of the percentage scale. However, the underlying distribution of actual severities may have ramifications for the number of classes needed on a scale to accurately estimate the mean disease severity (Hau et al. 1989). Kranz (1977) believed that the maximum likely severity to be encountered should be considered, and the scale set accordingly. However, it would not be appropriate to continually change the number of classes in a quantitative ordinal scale for every assessment. It will be best if the general conclusion for characteristics of quantitative ordinal scales and their design can be obtained by simulation. To this end, Chiang et al. (2014) and Liu et al. (2019) determined that a 10% interval scale with additional classes at <10% maximized accuracy of estimates of mean disease severity (Table 1). Unlike many other ordinal scales, this scale has a linear relationship with percentage area diseased at severities >10% (class 6 on the scale) (Fig. 2). Absolute errors (and standard deviations of the mean) of estimates tend to be least at severities less than approximately 10% for unaided raters—in the range where scale intervals are smaller (Hau et al. 1989; Bock et al. 2009b; Chiang et al. 2016a). The structures of the HB scale and some other quantitative ordinal scales discussed are presented (Table 1).

Nutter Jr. and Esker 2006) utilized a classical method (the difference threshold or just noticeable difference (JND)) developed in the field of psychophysics to evaluate the Weber-Fechner postulated properties of the HB scale. The basis of the HB scale is the so-called Weber-Fechner law. Weber’s law states that the physical size of a JND is a constant proportion of the value of the standard for a given dimension; Fechner’s law, which assumes Weber’s law, adds that the subjective value is a logarithmic function of the physical value. Nutter Jr. and Esker 2006) showed that raters more accurately discriminated disease severity between 25 and 50% compared to what the HB scale allowed. Furthermore, although Weber’s law holds true, Fechner’s law does not. The relationship between actual (true) disease severity and disease severity estimated by raters is linear, not logarithmic as proposed by the HB scale (Nutter Jr. and Esker 2006). Due to the relatively imprecise resolution of many ordinal scales, accurate estimates of severity using the 0–100% ratio scale (*i.e.*, NPEs) are almost always going to be more discerningly informative compared with those from an ordinal scale, *i.e.*, the structures of quantitative ordinal scale *versus* NPE being 1-to-*n*, where *n* is generally a small number (*e.g.*, *n* = 5) *versus n* = 100 for the percentage scale (Hartung and Piepho 2007; Bock et al. 2010a; Chiang et al. 2014).

The main questions of scale interval and structure are as follows: (i) What effects do equal or unequal intervals have on plant specimen disease severity estimates? (ii) How does interval width affect the estimates? and (iii) What is the optimal number of classes in a quantitative ordinal scale to maximize the accuracy of the estimates? Besides the nature of the quantitative ordinal scales, the calculation of the interval range is also considered. These questions need to be studied and understood to aid in the design of quantitative ordinal scales for those situations where they are to be preferred. What is the extent of inaccuracies caused by (i) structural elements of the ordinal scale (*i.e.*, equal or unequal classes, and the widths of the intervals), and (ii) using different methods to calculate the mean value (*i.e.*, midpoint or ordinal equivalent of the interval). Both factors might affect the estimated mean value and the variability of the resulting sample.

### The disease severity index

A special application of quantitative ordinal scales when estimating plant disease severity is the calculation of a disease severity index (DSI). The DSI is a single index number for summarizing a large amount of information on disease severity (Chester 1950; Chaube and Singh 1991). The disease severity is estimated by a rater as a value on the scale and has been used to determine a DSI on a percentage basis. When ordinal scale class rating is expressed in arbitrary grades, the formula for a DSI (%) can be written as follows:

1 $$ \mathrm{DSI}\ \left(\%\right)=\frac{\sum \left(\mathrm{class}\ \mathrm{frequency}\times \mathrm{score}\ \mathrm{of}\ \mathrm{rating}\ \mathrm{class}\right)}{\left(\mathrm{total}\ \mathrm{number}\ \mathrm{of}\ \mathrm{observations}\right)\times \left(\mathrm{maximal}\ \mathrm{disease}\ \mathrm{index}\right)}\times 100 $$

DSIs might be used to indicate the performance of a cultivar in regard to disease resistance at one location under a given set of conditions, to determine the effectiveness of fungicides, or to compare other treatments for disease control (Hunter and Roberts 1978; Kobriger and Hagedorn 1983; Kolkman and Kelly 2002; Kora et al. 2005; Gafni et al. 2015). Thus, studies in plant pathology, agronomy, and plant breeding often use a DSI based on a quantitative ordinal scale.

The DSI can convey some information to indicate the mean disease severity at a particular location, but is generally used to indicate mean disease severities in a single plot (replication, block, or tree, *etc.*). However, some previous work (Bock et al. 2010a; Chiang et al. 2014; Chiang et al. 2016a, 2016b; Bock and Chiang 2019) did not use the DSI to compare treatments, but used a sampling unit (*e.g.*, leaf, stem, and root) for comparing treatments. These approaches contrast in scale, but the same principles apply. Recently, Chiang et al. (2017a, b) explored the use of the DSI in regard to accuracy and hypothesis testing. The results are described in later sections.

### Methods of data analysis

Snedecor and Cochran (1989) and Madden et al. (2007) stated that standard parametric analyses including analysis of variance (ANOVA) may be appropriate for ordinal level measurements if the classes used in the scale represent equal intervals on an underlying continuous scale (for example, the percent scale). However, if data is based on an underlying (latent) continuous scale with unequal intervals between 0 and 100% (for example, the HB scale), ANOVA should not be used directly with the scale values; the recommended approach is to use the mid-point of the severity range for each class (Redman et al. 1969; Madden et al. 2007; Bock et al. 2010b; Chiang et al. 2014). Campbell and Madden (1990) emphasized that it is inappropriate to average the grade (or class) values because otherwise biased mean results would occur and negate any advantages of the disease assessment scale. Moreover, Chester (1950) stated that if the absolute disease severities of a class series are 0, 0^+^−3, 3^+^−8, 8^+^−20, 20^+^−50, 50^+^%, and if the classes are assigned arbitrary ratings of 0, 1, 2, 3, 4, 5, then indices of heavily diseased populations will not give a faithful representation of the more severe disease in comparison with populations having low severities (due to unequal interval sizes in the two scales, with lower severities having shorter interval sizes). It is reasonable to perform a midpoint conversion to ensure compatibility with parametric analysis (Bock et al. 2010b), but the mid-point value is an “approximation” to prevent excessive bias or loss of precision. However, in some cases, the mid-point of a class range will not be close to the actual severity owing to the wider interval size (*e.g.*, severities between 25 and 75% with the HB scale). In this regard, designing appropriate interval sizes is crucial.

Besides the midpoint of the interval being used, slightly different values may be selected based on the HB scale (Horsfall and Barratt 1945) by using the Elanco tables (Redman et al. 1969; Campbell and Madden 1990), but this approach has not been widely adopted. The geometric mean of the percentage assessment could be another choice as it is less distorted by an extreme individual score (Chaube and Singh 1991; Hartung and Piepho 2007). But Chiang et al. (2017a) showed that using the geometric mean in place of the midpoint value for each class did not improve the accuracy of estimates. In brief, the mid-point is preferable when a quantitative ordinal scale with unequal intervals is used, although it may not always be ideal.

Parametric proportional odds models may be used to analyze directly the class ratings obtained from quantitative ordinal scales (Chiang et al. 2020). These models were compared with midpoint conversions as well as with NPEs and results indicated that the performance of the proportional odds model is never inferior to using the midpoint conversion at severities <40%. Especially at low disease severities (≤10%), the proportional odds model was superior to using the midpoint conversion method. Thus, for early onset of disease, or for comparing treatments with severities <40%, the proportional odds model is preferable for analyzing disease severity data based on quantitative ordinal scales, and at severities >40% is equivalent to other methods. Proportional odds analysis is fairly complex relative to the other approaches mentioned (midpoint conversion followed by a t-test). If it is possible in the future, it will be useful to develop a package for combining a variety of approaches, including the proportional odds model, to provide plant pathologists with better means for determining improved methods of assessing and analyzing disease severity data.

## Applications and effects of quantitative ordinal scales

In general, there are two aspects to using a scale. First, the estimation (or prediction) that yields an absolute numerical value which may or may not be accurate. Second, when dealing with a comparison (using hypothesis testing) of two treatments, we must decide whether an observed difference can be attributed to chance. We review and discuss four cases according to the different purposes (estimation accuracy or hypothesis testing) and methods (empirical or simulated studies) which have been used to explore quantitative ordinal scales.

1. *Accuracy of severity estimates in empirical studies*

There has been a growing body of literature comparing different methods for estimating or determining the accuracy of disease assessment methods based on empirical studies. Since 2003, Lin’s concordance correlation (Lin 1989) has been widely used to quantify and compare the accuracy of estimates using different scale types (Nita et al. 2003; Bock et al. 2008a, 2008b; Bock et al. 2009c; Bock et al. 2013a, 2013b; Bardsley and Ngugi 2013). Nita et al. (2003) first used Lin’s concordance correlation analysis to determine estimation accuracy and reliability of estimates of Phomopsis severity on strawberry leaves and demonstrated that the HB scale was neither more accurate nor more reliable compared to NPEs. Bock et al. (2013b) showed that accuracy and reliability based on Lin’s concordance correlation depended on the range of actual severity and NPEs more often resulted in more precise and accurate estimates of pecan scab severity, particularly when estimating actual disease severities of 25^+^ to 75%, when compared to the HB scale.

Bock et al. (2013a) compared direct HB ratings and NPEs converted to HB scale ratings showing relatively minor effects on the proportion of incorrectly assigned categories. However, Forbes and Korva (1994) observed that in-field application of a quantitative ordinal scale (classes of 1–9) gave contrasting results to post-assessment conversion of NPEs to the ordinal scale. They concluded direct use of the ordinal scale resulted in a “linearizing” of the log-scale intervals, causing further bias in the mean value. Thus, raters estimated disease severities using ordinal equivalents as if all intervals were approximately equal (*i.e.*, the difference between 2 and 3 is similar to the difference between 3 and 4).

The focus of these studies is on the estimation of absolute numerical values for using rating scales. Empirical studies have suggested that HB scales and other quantitative ordinal scales are no better (and sometimes perhaps inferior) when compared with NPEs for assessing severity (Forbes and Korva 1994; Madden et al. 2007; Nita et al. 2003; Nutter Jr. and Esker 2006; Bock et al. 2009a). Indeed, Nita et al. (2003) concluded that a 5% increment scale was more accurate and reliable compared to the HB scale. Later, Hartung and Piepho (2007) analyzed rater assessments based on known distributions of disease severities to compare three different rating scales; the 0 to 100% ratio scale (NPEs), a 5%-step scale, and a quantitative ordinal scale with 9 classes, and found that NPEs were most precise but recommended a 5%-step scale due to simplicity and speed.

2. *Comparing treatments in empirical studies*

For treatment effects in planned experiments, hypothesis testing was used to explore the impact of using quantitative ordinal scales. Hypothesis testing requires that the data be sufficiently precise to reject the null hypothesis (H_0_ of no treatment effect), when H_0_ is false, or conversely, to accept H_0_ when there are no treatment differences. Failure to reject H_0_ when H_0_ is false results in commission of a type II error, while rejection of H_0_ when H_0_ is true results in commission of a type I error. The power of the hypothesis test, *i.e.*, when the probability of rejecting Ho when Ho is false, depends on the type II error rate (power = 1 − type II error rate).

Visual assessments may fail to discern differences among treatments where more objective (*i.e.*, more accurate, precise, and reproducible estimates of the area covered by symptoms on a leaf, plant, or in a crop) measures of severity show a significant effect (*i.e.*, a type II error). Todd and Kommedahl (1994) compared image analysis (0 to 100%) and quantitative ordinal scale (0 to 4, Table 1) estimates to evaluate of Fusarium stalk rots in corn. Image analysis provided a more precise measure of severity compared to the scale ratings.

For phenotypic evaluations in quantitative genetic studies, Xie et al. (2012) investigated the application of image analysis to study common bacterial blight resistance in common bean. Their results indicated that the visual assessment results (based on a 0 to 5 scale, see Table 1) overestimated the effect of quantitative trait locus (QTL) in genetic studies (which may have been caused by lack of additivity and the unequal intervals of the ordinal scale). It further demonstrated that severity measurements using image analysis were not linearly associated with visual estimates directly using a non-linear 0 to 5 scale, resulting in error. Most often there is a linear relationship between image analysis measurements of disease severity and visual estimates on the percentage scale (Nutter Jr. et al. 1993; Nutter Jr. and Esker 2006; Bock et al. 2008a). Poland and Nelson (2011) also observed less precision in estimates using a 1 to 9 scale (Table 1) compared using the percentage scale but, in this case, did not affect the identification of QTLs.

Bock et al. (2015) investigated the effects of rater accuracy and assessment methods for comparing treatments on disease severity estimates. In that study, NPEs of Septoria leaf blotch (SLB) on leaves of winter wheat from nontreated and fungicide-treated plots were assessed. NPEs were converted to HB scale midpoints. All rater estimates differentiated treatments; but conversion of NPEs to the HB scale reduced F values slightly, and in one case means separation was different. Collectively, these studies indicate that the accuracy and reliability of estimates are affected by rater and assessment methods used, and these can affect the mean estimates and the outcome of means comparisons.

3. *Accuracy of severity estimates using simulation studies*

The first study to use simulation to investigate aspects of disease assessment was that of Forbes and Korva (1994). They explored the effects of scale type on the distribution of estimates. NPEs of disease severity were compared to the use of a quantitative ordinal scale, showing that NPEs were superior. Chiang et al. (2017a) investigated the process of calculating the DSI on a percentage basis from quantitative ordinal scale data and explored the effects of both different scales types (equal or unequal intervals) and calculation methods (score or midpoint) of the intervals on the accuracy of the DSI estimates, with the aim of developing a framework to maximize accuracy (*i.e.*, minimize the deviations from the actual severity) of data if using a DSI. A simulation approach was developed to explore the effect of using different methods (interval midpoint or the ordinal equivalent) for calculation of the interval range and the nature of the ordinal scales (equal or unequal intervals) used for the DSI estimates (%). The objective was to determine the conditions for maximizing the accuracy of estimation of the actual mean value based on a DSI. That is, the study dealt with the issue of how to determine a specific, not a comparative, value (*i.e.*, which method is the most accurate rather than comparing two values for the purpose of hypothesis testing). If rater estimates were unbiased, compared with other methods tested, the most accurate method for estimation of a DSI is to use the midpoint of the severity range for each class with an amended 10% ordinal scale (a quantitative ordinal scale based on a 10% linear scale, with additional grades at low severities (Chiang et al. 2014)). In a situation where a rater is biased, the accuracy of the resulting DSI estimates (%) will depend mainly on the degree and direction of the rater bias relative to the actual mean value.

Recently, Liu et al. (2019) used simulation and actual data of mean severity of pear scab to compare mean estimates based on various quantitative ordinal scale designs to those based on NPEs, and to investigate the effects of the number of classes in an ordinal scale on the accuracy of that mean. The results indicated that scales with seven or more classes are preferable when actual mean disease severities of ≤50% are involved. Moreover, the use of an amended 10% quantitative ordinal scale with additional classes at low severities resulted in a more accurate mean severity compared to most other scale designs at most mean disease severities (developed by Chiang et al. 2014, Table 1).

A recent article describing clinical research on COVID-19 (WHO Working Group on the Clinical Characterisation and Management of COVID-19 infection 2020) also embraced the notion for the design of disease scales to emphasize low severities. They stated that “Modelling in other disease states has shown that distinction is greater when seven or more classes are used, particularly at the lower range of disease severity.” Despite the WHO clinical COVID-19 progression scale being a qualitative ordinal scale (for which numeric means are somewhat “biologically meaningless”), the point of discussion is in regard to the importance of low severity cases.

Quantitative ordinal scales are now being packaged in apps to facilitate disease severity assessment. Two diagrammatic-based quantitative ordinal scales are available in the Estimate app used to improve the accuracy of estimates of Cercospora leaf spot of table beet (Pethybridge and Nelson 2018). One is based on a linear system, the other on an HB logarithmic system. Using either system the first stage allows the rater to select a class for the specimen, and a second stage allows the rater to select a severity within that class interval range. Del Ponte et al. (2019) found that using the two-stage linear scale option was most accurate when compared to using the equivalent two-stage HB scale option for assigning a severity value to a specimen.

4. *Comparing treatments using simulation studies*

**Fig. 3:**
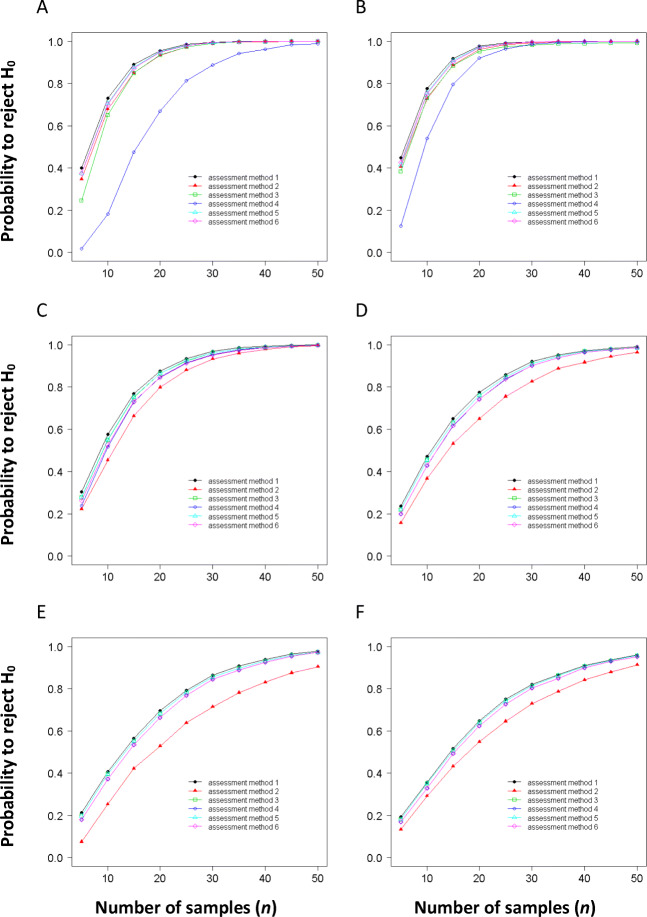
The relationships between the probability to reject H_0_ (when this hypothesis is false) and samples size (*n* = 5–50) for the different assessment scales at different population means (**A**, 1; **B**, 5; **C**, 20; **D**, 30; **E**, 40; and **F**, 50% disease severity). The difference between the population means (μ_△_) is assumed to be 10%, with significance at *P* = 0.05. *φ* (= 5%) represents that the standard deviations of the disease severity distributions of treatment A and B are assumed to be equal. Assessment method 1: NPEs; method 2: HB scale; method 3: linear scale (5%); method 4: linear scale (10%); method 5: amended linear scale (5%); and method 6: amended linear scale (10%). Figure from Chiang et al. (2014)

During the last 10 years, simulation has been used to probe hypothesis testing for treatment effects in randomized replicated experiments, thereby providing a basis to compare the different assessment methods quantitatively (Bock et al. 2010a; Chiang et al. 2014). Bock et al. (2010a) set the precedent for comparing quantitative ordinal rating scales (the HB category scale) to NPEs using data from citrus canker-infected grapefruit leaves (Bock et al. 2008a, 2008b) with actual disease severity ranging from 0 to 50%. The results indicated that the HB category scale was never better than NPEs for comparing treatments, and the HB scale could result in less precise data, elevating the risk of a type II error. Subsequently, Chiang et al. (2014) applied the same approach to explore interval characteristics that would result in an optimally designed quantitative ordinal scale. The results showed that an amended 10% category scale (10% linear scale emphasizing severities ≤50% disease, and with additional intervals at severities <10%) is superior to other quantitative ordinal scales for reducing the risk of type II errors and for raters who desire to base their severity estimation on a quantitative ordinal scale for hypothesis testing. Moreover, the mixed model and the bootstrap analyses both confirmed the results of the simulation studies. In the article by Chiang et al. (2014), NPEs, HB midpoint data, and four other linear quantitative ordinal scales were compared (5% and 10% increments, with and without additional grades at low severity). The conclusion was that a quantitative ordinal scale should be sensitive to low disease severity (1–10%) by incorporating additional classes to account for disease severity ≤5% and that intervals in the mid-range should not exceed 10%. Moreover, scales with equal interval widths (5 or 10%) tend to have a greater risk of type II error at low disease severities (≤5%) (see Assessment methods 3 and 4, Fig. 3A). The results contrast a little with those of Hartung and Piepho (2007), who recommended that a 5%-step scale be used, although 1%-step scale was slightly more precise (for practical reasons they preferred the 5%-step scale). The tendency to overestimate is most consistent and proportionately greatest (based on relative error) at diseases severities <10% (Sherwood et al. 1983; Bock et al. 2008b), although it is also common based on the mean estimate (Nutter Jr. et al. 1993; Forbes and Korva 1994; Parker et al. 1995a; Godoy et al. 1997; Nita et al. 2003). For many studies in plant pathology, it is a disease at low severity that needs to be most accurately estimated because these assessments form the basis for estimating parameters that might be used in projecting epidemic development, or comparing treatments of most interest.

Neither Bock et al. (2010a) nor Chiang et al. (2014) considered how over- or underestimation (bias) might impact hypothesis testing. As noted, there is a tendency to overestimate disease severity at low actual severities (*<*10%) (Sherwood et al. 1983; Bock et al. 2010b), particularly when the disease was characterized by numerous small lesions, as confirmed by Forbes and Jeger (1987) (estimation of severity with fewer larger lesions is less error-prone). Although the effect of bias is well-recognized (Amanat 1976; Sherwood et al. 1983; Beresford and Royle 1991; Forbes and Korva 1994; Bock et al. 2008b and 2009b), the impact of bias on hypothesis testing across the range of severity, and the effects of scale type on bias was only recently explored (Chiang et al. 2016a). The results demonstrated the power of the hypothesis test is greatest when estimates are unbiased, yet neither rater bias nor assessment method inflates type I errors. That is, the power of the test is reduced yet further with biased measurements of severity unless the bias is uniform for all levels of severity, but this case is hard to achieve. An unanticipated but important observation was the greater impact of rater bias compared with the assessment method on type II errors. NPEs and the amended 10% category scale with additional grades at low severity most often had the lowest type II error rates compared to other quantitative ordinal scales tested (Chiang et al. 2016a). These observations reinforce how essential it is to reduce bias in rater estimates in order to improve accuracy and reliability of visual estimates of disease severity, and thus avoid type II errors in the analysis of disease severity data (Christ 1991; Todd and Kommedahl 1994; Parker et al. 1995a).

**Fig. 4:**
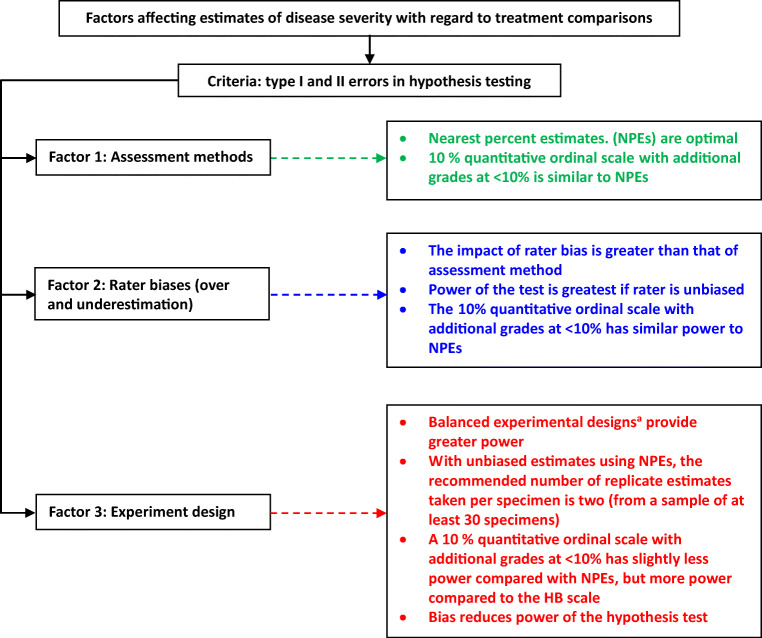
A flowchart providing an overview of characteristics of scale factors affecting treatment comparisons, with emphasis on quantitative ordinal scales. ^a^Balanced experimental designs: the same number of samples for each treatment group

It is important to consider resource use efficiency (labor, time, and money) when selecting scale type and experimental design (Chiang et al. 2016b). Some medical studies have investigated how funding constraints determine the recruiting cost of specimens needed for reliability studies (Shoukri et al. 2003; Shoukri 2004). That is, the objective of the studies is to collect reliable data for testing purposes (so the need is to consider reliability and cost—selecting the number of specimens and replicate (or sub-samples) estimates per sample to ensure sufficient reliability or agreement, while minimizing cost). Bousset et al. (2016) also pointed out that, with given limited resources, cost is an important consideration in plant pathological studies. Chiang et al. (2016b) presented the concepts applied in medical research (Giraudeau and Mary 2001; Shoukri et al. 2003; Shoukri 2004) and concluded that developing an optimal experimental design in which the number of specimens (individual units sampled) and the number of replicates (sub-sample) estimates per specimen for a fixed total number of observations (total sample size for the treatments being compared) are chosen to maximize statistical power and efficiency. Chiang et al. (2016b) indicated, with unbiased estimates using NPEs, the recommended number of replicate estimates taken per specimen is two (from a sample of specimens of at least 30), as this conserves resources for a given mean, mean difference of treatments, distribution of observations, and variances. It is best to avoid using an unbalanced experiment design, particularly when combined with the HB scale method of assessment (or other quantitative scales with similarly unequal intervals). A balanced experimental design is one with the same number of samples for each treatment group. The power of the test is reduced yet further if the rater has a tendency to biased estimates, as described above. These are general recommendations. An overview of factors affecting the assessment of disease severity with regard to treatment comparisons is presented (Fig. 4).

**Fig. 5:**
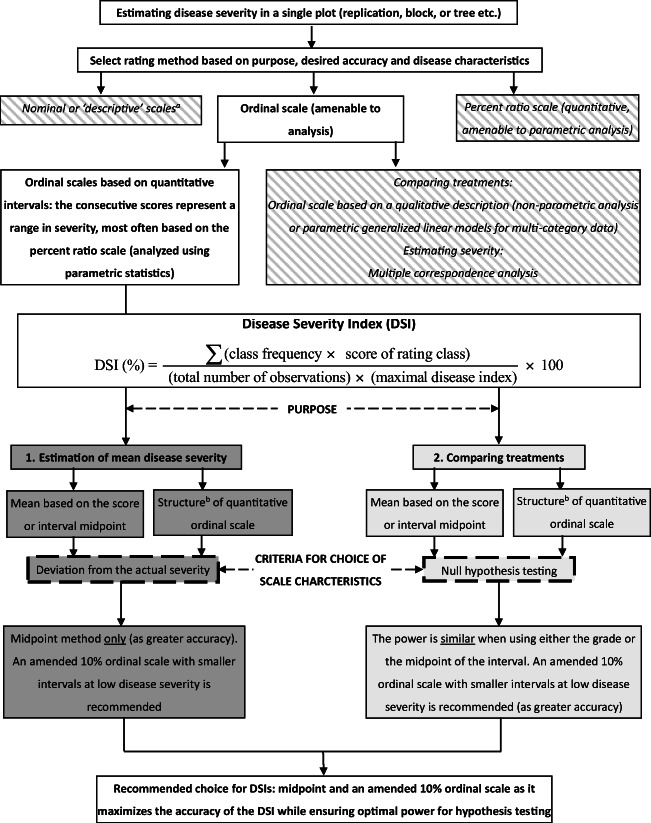
A flowchart providing an overview of the options for assessing disease severity, and specifically the process used for establishing a quantitative ordinal scale using a disease severity index (DSI) regarding the number of grades, the nature of the grade values, and the method (interval midpoint or grade values) for estimating mean disease severity and/or hypothesis testing. The results demonstrated that an amended 10% category scale (10% linear scale emphasizing severities ≤50% disease, and with additional intervals at severities <10%) provided both accuracy for estimating the disease severity, and optimal power for hypothesis testing (Chiang et al. 2014 and 2017b). The boxes with the parallel gray bars indicate other methods for assessing disease severity, depending on the purpose, desired accuracy, and disease characteristics. Solid dark gray boxes indicate the process of DSI scale selection for estimating only the disease severity, while the pale gray boxes indicate the process of DSI selection for hypothesis testing. Figure from Chiang et al. (2017b). ^a^ Nominal scales are qualitative in nature. If only two states (healthy *vs.* diseased), it constitutes an incidence measure. If >2 descriptors it may define a range of severity *e.g.*, “light,” “moderate,” and “severe” or indicated as “+,” “++,” “+++.” There are methods for dealing with disease incidence data described elsewhere (*e.g.*, Madden et al. 2007). ^b^ *i.e.*, interval width(s), and equal or unequal scale intervals

As noted, the DSI can be used for summarizing quantitative ordinal scale data. Chiang et al. 2017explored the impact of using different quantitative ordinal scales to estimate the DSI for comparing treatments with hypothesis testing. The results demonstrated that both quantitative ordinal rating classes or the midpoint conversion for the ranges of those classes had similar power. However, the principal factor determining the power of the hypothesis test is the structure of the scale intervals, not the method of value selection for scale intervals (*i.e.*, either the mid-point or ordinal equivalent). To provide guidance on choices, we have presented a flowchart for reference in selecting scales and values for scale intervals when using a DSI (Fig. 5).

## Concerns regarding use of quantitative ordinal scales for validation of sensor-based measurement

We have discussed the application and design of quantitative ordinal scales for estimating the severity of plant diseases for direct use in research. Because quantitative ordinal scales are based on grouping of discrete units, they can be used as a basis to classify the accuracy of measurements from imaging systems including visible spectrum, hyperspectral or multispectral sensors. In these cases, the ordinal scale rankings may be considered surrogates (and groupings) for the actual, or true values (a process known as ground-truthing in remote sensing).

Visible spectrum (VIS) image analysis has the potential to be accurate, repeatable, and reproducible (Martin and Rybicki 1998; Bock et al. 2008a; Bock and Nutter 2011; Barbedo 2014, 2016, 2017; Barbedo 2019). However, the evaluation of measurements obtained using VIS image analysis is not straightforward. The systems developed are dependent on the references they are tasked to mimic (Bock et al. 2020). In some cases, using VIS image analysis, the classifiers require the measured severity to be transformed from continuous data to a discrete scale of values, and measurements compared to a limited number of classes on a quantitative ordinal scale, resulting in lower resolution of severity differentiation compared to the percentage scale. For example, Esgario et al. (2020) used 5 classes for severity ranges assigned as follows: healthy (< 0.1%), very low (0.1−5%), low (5.1−10%), high (10.1−15%), and very high (> 15%). If a quantitative ordinal scale is used as a surrogate for actual values, it causes the information to be distorted in the process of transforming NPE data into ordinal disease severity scores. Thus, it increases inaccuracy of the mean value and variance of the sample mean, which will elevate the risk of type II errors (Chiang et al. 2014; Chiang et al. 2016a, 2016b). If the resulting data were to be used in the analysis, there would be an increased risk of failing to reject the null hypothesis when it is false.

Similarly, quantitative ordinal scales have been used to ground-truth hyperspectral and multispectral imaging (HSI and MSI) of plant disease severity (Coops et al. 2003; Huang et al. 2007; Leucker et al. 2016; Mahlein 2016; Wang et al. 2016). For example, several studies have explored classification accuracy using ordinal groupings in classes of visually assessed specimens as the assumed ground-truth values (Bravo et al. 2003; Alisaac et al. 2018; Thomas et al. 2018; Alisaac et al. 2019; Bock et al. 2020).

Quantitative ordinal scale disease severity estimates based on visual assessments may be used alongside automated sensor systems for ground-truthing when developing new or improved fully automated artificial intelligence-based methods. The use of actual or ground-truth values in all studies is critical to the ongoing process of ensuring the accuracy of estimation and for maximizing the statistical power of subsequent hypothesis tests. When a quantitative ordinal scale is being used for ground-truthing or validation, it is crucial to identify its structure (*i.e.*, does the scale have equal or unequal intervals? and what is(are) the interval width(s)?). If a quantitative ordinal scale design is optimal for visual assessment with regard to accuracy and maximizing the power of hypothesis tests, values expressed using the scale will also be more accurate and reliable when compared to the results of sensor-based image analysis.

The characteristics of quantitative ordinal scales such as those used for sensor-based image analysis have not been described sufficiently to make possible a full understanding of the ramifications of using them. There are very few investigations into a sensor-based measurement that have considered the ramifications of using a quantitative ordinal scale for validation. We believe that the further development of quantitative ordinal scales for use in visual estimates can provide some valuable information for the validation of sensor-based measurement. That is, such development can be used to quantify image analysis using visible spectrum, hyperspectral, or multispectral sensors for detecting and measuring plant diseases.

## Quantitative ordinal scales—some future research needs

Ordinal scales remain widely used, perhaps with limited regard for potential consequences if they are misused or poorly designed. There remain several aspects of ordinal scales and their applications that are poorly understood. Further research is needed. These are a few areas for consideration.

1. Time. One study indicated quantitative ordinal severity scales saved time (Hartung and Piepho 2007), yet a second failed to demonstrate a time advantage (Bock et al. 2013a). Faster assessments may be less precise (Parker et al. 1995b). Furthermore, more replicates are needed to maintain the same power when using a quantitative ordinal scale compared to NPEs, and those additional replicates will take time to procure (Nutter Jr. and Esker 2006; Bock et al. 2010a, 2010b; Chiang et al. 2014). A definitive study to demonstrate time advantage without loss of power is needed.
2. Conversion to NPEs. In many studies, NPE data were directly converted to the appropriate interval on the quantitative ordinal scale and thence converted to the appropriate midpoint value of each grade for analysis to compare the different assessment methods regardless of whether the study was empirical or a simulation (Nita et al. 2003, Bock et al. 2008a, 2008b, Bock et al. 2009a, Bock et al. 2013a, 2013b; Chiang et al. 2014, Chiang et al. 2016a, 2016b, Chiang et al. 2020). Direct application of quantitative ordinal scales by raters has been studied on only a few occasions (Bock et al. 2013a, 2013b; Forbes and Korva 1994; O’Brein and van Bruggen 1992), and incorrect assessment of disease severity can lead to additional error (Kranz 1977). To aid best scale development, a better understanding is needed between direct use of different quantitative ordinal scales and relationships with the actual severity values.
3. For methods of calculating the severity interval of quantitative ordinal scales, the interval is traditionally replaced by mid-point imputation or by the class number of the interval. However, such interval-based data may not be precise enough. In this situation, where we know only that a value is within a particular range, the data may be interval-censored (Onofri et al. 2019). The body of techniques designed to deal with censoring is known as “survival analysis” (Klein and Moeschberger 2003; Kleinbaum and Klein 2012). Survival analysis uses all available information within a parametric modeling framework and takes into account the uncertainty due to censoring. Recently, Onofri et al. (2019) stated that methods of survival analysis for censored data can be useful, leading to reliable inferences and sound hypotheses testing. Survival analysis might be compared to other methods for comparison of treatments using quantitative ordinal estimates of disease severity.
4. Do standard area diagrams (SADs) allow for more accurate use of quantitative ordinal scales (*i.e.*, reduce misclassifications into classes or grades)? Standard area diagrams are demonstrated to provide more accurate estimates of severity using the percentage scale (Del Ponte et al. 2017), but do they have the same effect for ordinal scale estimates? If so, should they be designed to relate to the class values or midpoints of ordinal scales? Would generic SADs be sufficient, or would each pathosystem require its own SADs?
5. Most studies have simulated average raters using quantitative ordinal scales. Understanding the ramifications of raters with different precision and accuracy (with and without bias) will deepen our understanding of penalties due to inaccuracy and impacts on hypothesis testing where raters of different abilities are concerned.

## Conclusion

Plant pathologists face many situations where estimates of disease severity using NPEs may be too time-consuming or impractical. In such situations, researchers have often used a quantitative ordinal scale for measurement (Bock et al. 2010b; Chiang et al. 2020). If an “optimal” scale design can be adopted to measure disease severity, it will be of considerable practical value in many arenas where disease severity data is required. Based on some published results (Bock et al. 2010a; Chiang et al. 2014), a quantitative ordinal scale should be designed with sensitivity to low disease severity to prevent potential overestimation; categories of 0.1, 0.5, 1.0, and 5.0% might fulfill this requirement. Interval ranges for classes at severity ≥10% should not exceed 10%. A scale with these characteristics has been named “an amended 10% ordinal scale” as proposed by Chiang et al. 2014, Chiang et al. 2016a, 2016b) and Bock et al. (2020). The “Chiang” scale had almost equal ability to NPEs in the accuracy of the mean and power to compare treatments. Discipline-wide standards should be considered for optimizing quantitative ordinal scale design to ensure accuracy of disease severity estimation. A novel and valuable insight from recent research was that taking two replicate (sub-sample) estimates per specimen (from a sample of at least 30 specimens [individual units sampled]) will increase the efficiency of resource use while maintaining the same statistical power as that obtained for a total sample size of 120 specimens for both treatments (Chiang et al. 2016b). Unbalanced experimental design should be avoided, particularly when used in combination with quantitative ordinal scales with uneven intervals. These general recommendations are applicable in most, or at least many, plant disease assessment situations.

## Data Availability

Data sharing not applicable to this article as no datasets were generated or analyzed during the current study.
